# Preparation of a g-C_3_N_4_/UiO-66-NH_2_/CdS Photocatalyst with Enhanced Visible Light Photocatalytic Activity for Tetracycline Degradation

**DOI:** 10.3390/nano10091824

**Published:** 2020-09-12

**Authors:** Hao Zhang, Jialiang Li, Xianglei He, Bo Liu

**Affiliations:** 1 School of Chemistry and Chemical Engineering, Shandong University of Technology, Zibo 255000, China; haozhangy@163.com (H.Z.); xianglei_he@163.com (X.H.); 2 Laboratory of Functional Molecules and Materials, School of Physics and Optoelectronic Engineering, Shandong University of Technology, Zibo 255000, China

**Keywords:** photocatalytic, g-C_3_N_4_, composite, tetracycline

## Abstract

A combination of calcination and hydrothermal processing was used to prepare a g-C_3_N_4_/UiO-66-NH_2_/CdS photocatalyst, and the degradation of tetracycline (TC) over this material was assessed. The photocatalytic performance of this nanocomposite was approximately 4.4 and 2.3 times those of CdS and g-C_3_N_4_, respectively, and was found to be affected by the CdS loading amount, the pH of the reaction solution and the initial TC concentration. This catalyst also exhibited stable performance over four consecutive reaction cycles. The highly enhanced photoactivity of the g-C_3_N_4_/UiO-66-NH_2_/CdS is attributed to the introduction of CdS, which widens the range over which the material absorbs visible light and inhibits the recombination of electron–hole pairs. The results of this study suggest further applications for this material in the treatment of contaminated wastewater powered by solar energy.

## 1. Introduction

Environmental pollution by antibiotics is a pressing global problem. Because these compounds are resistant to degradation, they are not effectively removed by traditional water purification treatments, and so represent a potential threat to human health and the environment. It would, therefore, be beneficial to develop an effective means of removing antibiotic pollutants. To date, the most common methods of treating wastewater containing antibiotics include biodegradation [1], membrane separation [2], adsorption [3], and photocatalysis [4]. Among these, photocatalysis has been most widely studied because this process is both environmentally friendly and simple.

Graphitic carbon nitride (g-C_3_N_4_), a metal-free photocatalyst with a two-dimensional (2D) structure, has the advantages of being easy to synthesize, highly stable, inexpensive, and nontoxic [5]. For these reasons, this material has been widely used in photocatalysis, including applications such as water splitting [6], the degradation of organic compounds [7], the reduction in Cr (VI) [8], and the conversion of CO_2_ [9]. Even so, because g-C_3_N_4_ exhibits minimal absorption of visible light and poor charge transport characteristics, its photocatalysis performance is generally unsatisfactory [10]. To date, a variety of strategies for improving the performance of g-C_3_N_4_ have been developed, such as doping with heteroatoms [11], the formation of composites with other semiconductors [12], and dye-sensitization [13]. Among these methods, the fabrication of composite materials has been found to greatly increase not only the visible light utilization efficiency of g-C_3_N_4_ but also the separation rate of photogenerated electron–hole pairs. Hence, various g-C_3_N_4_-based visible-light-driven composite photocatalysts have been reported, including g-C_3_N_4_/metal organic frameworks (MOFs) [14], g-C_3_N_4_/CdS [15], and g-C_3_N_4_/AgPO_3_ [16].

More recently, ternary g-C_3_N_4_/MOF/X photocatalytic systems have become a research focus because these materials provide interfaces with a high degree of contact along with synergistic effects based on their various components [17]. As an example, Wang et al. [18] combined UiO-66 as a cocatalyst with N-K_2_Ti_4_O_9_/g-C_3_N_4_ to promote the degradation of Rhodamine B (RhB) under visible light. Their experimental data suggested that the resulting increase in photocatalytic activity could be attributed to the greater adsorption capacity and the synergistic effect derived from the combined ingredients. Liang et al. [19] prepared BiOI@UiO-66(NH_2_)@g-C_3_N_4_ via an in-situ solvothermal–hydrothermal method, and showed that this Z-scheme photocatalyst exhibited improved activity during the degradation of RhB or tetracycline (TC) under visible-light. Liang’s data also demonstrated that the enhanced photocatalytic performance was caused by the introduction of the BiOI, which widened the photoabsorption range of the catalyst and promoted the formation of an n-p-n type heterojunction to drive the Z-scheme mechanism. Among the materials assessed thus far, CdS has been especially widely investigated, because it provides excellent visible-light response and a suitable band width of about 2.4 eV while being simple to prepare.

In the present paper, we synthesized a g-C_3_N_4_/UiO-66-NH_2_/CdS composite photocatalyst and characterized this material using X-ray diffraction (XRD), fourier transform infrared (FT-IR) spectroscopy, scanning electron microscopy (SEM), and transmission electron microscopy (TEM). Using TC as a model pollutant, photodegradation experiments were performed to evaluate the photocatalytic performance of this composite. The effects of CdS loading, the initial TC concentration, the pH of the reaction solution, and the other factors on the photocatalytic activity were studied. The results show that this as-synthesized g-C_3_N_4_/UiO-66-NH_2_/CdS composite material effectively degraded TC in water, suggesting that this material has potential as a photocatalyst.

## 2. Materials and Methods

### 2.1. Materials

Zirconium chloride (ZrCl_4_, 99.9%), cadmium nitrate (Cd(NO_3_)_2_, 99%), urea (CO(NH_2_)_2_, 99%), 2-aminoterephthalic acid (H_2_BDC-NH_2_, 98%), thiourea (CH_4_N_2_S, 99%), polyvinyl pyrrolidone (PVP, 99%), ethanol (C_2_H_5_OH, 99.5%), methanol (CH_3_OH, 99.5%), N,N-dimethylformamide (DMF, 99.5%), sodium hydroxide (NaOH, 96%), hydrochloric acid (HCl, 37wt%), and TC (C_22_H_24_N_2_O_8_, 99%) were purchased from the Sinopharm Chemical Reagent Co., Ltd. (Shanghai, China). All chemicals were obtained commercially and used without further purification.

### 2.2. Preparation of g-C_3_N_4_

The required amount of urea was ground and transferred to a crucible with a lid. The crucible was then placed in a muffle furnace and heated from ambient temperature to 550 °C at 5 °C/min, then maintained at that temperature for 4 h in air. After cooling the product, it was ground to obtain g-C_3_N_4_ as a light-yellow powder.

### 2.3. Preparation of g-C_3_N_4_/UiO-66-NH_2_ Composite

A 3:1 (by mass) mixture of g-C_3_N_4_ and UiO-66-NH_2_ was ground for 30 min, then transferred into a crucible and heated in a muffle furnace at 350 °C for 2 h in air. The g-C_3_N_4_/UiO-66-NH_2_ composite was collected after cooling and is referred to herein as gU-3.

### 2.4. Preparation of the g-C_3_N_4_/UiO-66-NH_2_/CdS Composite

**Figure 1:**
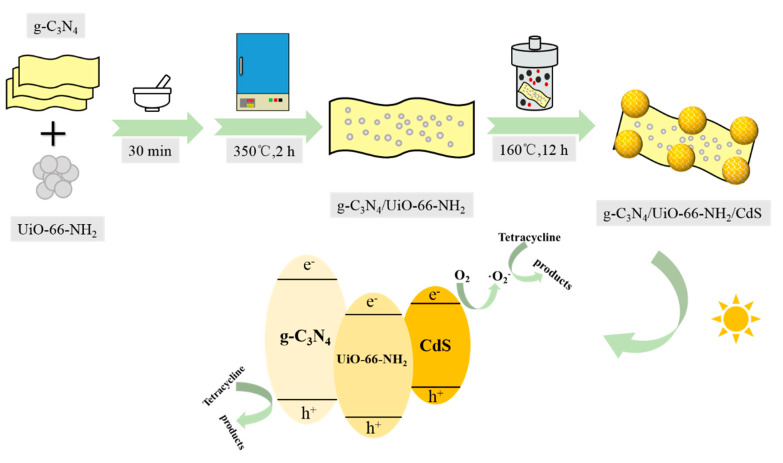
Schematic illustration of the synthetic route to the g-C_3_N_4_/UiO-66-NH_2_/CdS composites.

Cd(NO_3_)_2_ (7.5 mmol) and thiourea (7.5 mmol) were dispersed in deionized water (50 mL) after which PVP (90 mg) was dissolved in the mixture. Various amounts of gU-3 were added to this solution, followed by ultrasonication for 1 h and stirring at 1200 rpm for 6 h. The resulting mixture was transferred to a 100 mL hydrothermal reactor and then heated in an oven at 160 °C for 12 h. The sample was allowed to cool and the product was collected by centrifugation at 8000 rpm, washed with H_2_O and ethanol to remove impurities, and finally dried at 80 °C under vacuum for 10 h to give the g-C_3_N_4_/UiO-66-NH_2_/CdS. The materials synthesized in this manner are referred to herein as gUS-X, where X = M_CdS_/M_gU-3_. Figure 1 presents a diagram summarizing the synthesis of these composites.

### 2.5. Characterization

The crystalline structures of the g-C_3_N_4_/UiO-66-NH_2_/CdS photocatalysts were assessed by XRD using a Bruker D8-ADVANCE (Bruker AXS, Karlsruhe, Germany) with Cu Kα radiation over the 2θ range of 5–60°. FTIR spectra (KBr pellets as substrate) were recorded on a Nicolet 5700 spectrophotometer (Thermo Fisher Scientific, Waltham, MA, USA) in the range from 4000 to 400 cm^−1^. The morphologies of the photocatalysts were examined by TEM and SEM using a Tecnai G2 F20 S-TWIN (FEI, Hillsboro, OR, USA) and Sirion 200 (FEI, Hillsboro, OR, USA). Photoluminescence (PL) spectra were acquired using a fluorescence spectrophotometer (F-380, Hitachi, Tokyo, Japan). Ultraviolet-visible (UV-Vis) absorption spectroscopy and diffuse reflectance spectroscopy (DRS) were performed using a spectrophotometer (UV-3600, Shimadzu, Kyoto, Japan) over the range of 200–800 nm. An ESCALAB 250XI (Thermo Fisher Scientific, Waltham, MA, USA) with a He I (21.22 eV) lamp was employed to perform UV photoelectron spectroscopy (UPS) to ascertain the valence band positions and work functions of the samples. Electrochemical impedance spectroscopy (EIS) was conducted with an electrochemical workstation (CHI 760E, CH Instruments, Shanghai, China) using a standard three-electrode system. The pH values of the various samples were determined using a pH meter.

### 2.6. Evaluation of Photocatalytic Activity

In a typical trial, 50 mg of the g-C_3_N_4_/UiO-66-NH_2_/CdS photocatalyst was suspended in 100 mL of a 20–50 mg/L TC solution, and a full spectrum 300 W xenon lamp was used as the light source. Prior to illumination, the reaction mixture was stirred in the dark for 60 min to ensure adsorption–desorption equilibrium between the TC and photocatalyst, after which irradiation was applied. During the exposure to light, 6 mL aliquots of the suspension were extracted at 30 min intervals for 180 min and centrifuged. UV–Vis spectra of the supernatants were then acquired to determine the residual concentration of TC. The pH of the TC solution was regulated using 0.05 M NaOH or HCl solutions before the start of the reaction. The TC photodegradation efficiency (D%) was calculated as:

(1) $$D\%=\frac{C_{0}-C_{t}}{C_{0}}\times 100\%$$

where *C*_0_ is the initial TC concentration and *C_t_* represents the residual concentration of TC after a specific irradiation time (*t*).

## 3. Results and Discussion

### 3.1. Characterization of the g-C_3_N_4_/UiO-66-NH_2_/CdS Composite

**Figure 2:**
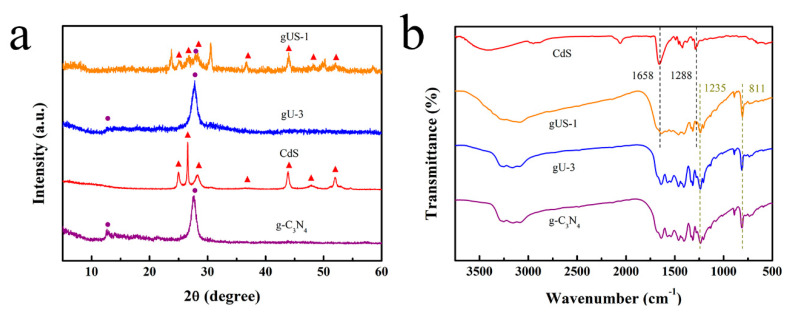
(**a**) XRD patterns and (**b**) FTIR spectra of the various photocatalysts.

The XRD patterns obtained from the as-prepared gUS-1 and gU-3 and from the original CdS and g-C_3_N_4_ are shown in Figure 2a. The pattern produced by the g-C_3_N_4_ is consistent with literature reports and contains two characteristic peaks at 12.7° and 27.5° corresponding to the (100) and (002) planes of the graphite phase structure, respectively [20,21]. The pattern generated by the g-C_3_N_4_/UiO-66-NH_2_ (gU-3) is similar to that of the g-C_3_N_4_, indicating that the crystal structure of the latter was not changed after the addition of the UiO-66-NH_2_. The lack of an effect can possibly be ascribed to the low loading levels and high dispersion of the UiO-66-NH_2_ in the composite of gU-3. In the case of the CdS, the XRD pattern shows a hexagonal phase, and the main diffraction peaks appear at 25.3°, 26.7°, 28.3°, 37.2°, 43.9°, 48.2°, and 52.1°, corresponding to the (100), (002), (101), (102), (110), (103), and (201) crystal planes, respectively [22]. Interestingly, all these peaks were also produced by the g-C_3_N_4_/UiO-66-NH_2_/CdS composite (gUS-1), suggesting that the crystal structure of the CdS was well-preserved after this compound was anchored onto the g-C_3_N_4_/UiO-66-NH_2_ sheets. Additionally, the gUS-1 hybrids showed new peaks at 23.7° and 30.5° that correspond to donor-acceptor interactions between the CdS and g-C_3_N_4_ [23,24].

The FTIR spectra of the g-C_3_N_4_, gU-3, gUS-1, and CdS are presented in Figure 2b. The g-C_3_N_4_ spectrum exhibits peaks at 811 and 1235 cm^−1^ that are ascribed to the bending vibration of triazine units and stretching modes of C–N–C groups [25]. The CdS also generated peaks between 1200 and 1800 cm^−1^ that are attributed to Cd-S bonds [26]. Interestingly, the spectra of the gU-3 and gUS-1 are similar and both contain a peak related to g-C_3_N_4_, confirming that g-C_3_N_4_ was the primary component of both. In addition, the two characteristic CdS peaks at 1288 and 1658 cm^−1^ are present in the gUS-1 spectrum, indicating the successful loading of CdS in the gUS-1.

**Figure 3:**
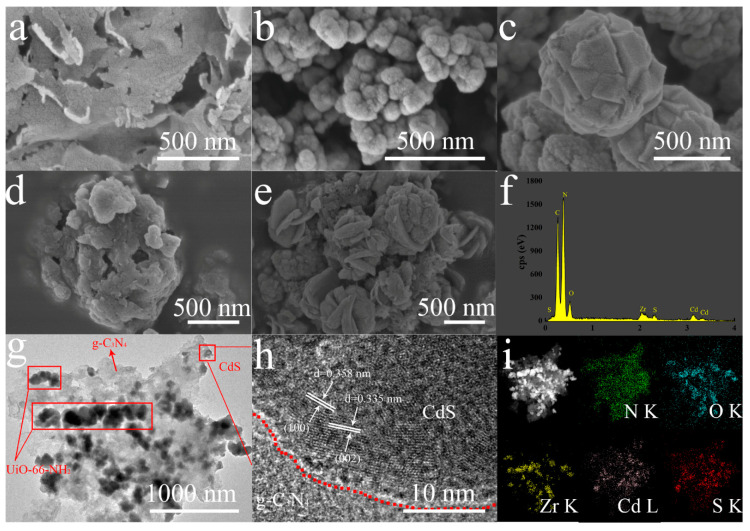
SEM images of the (**a**) g-C_3_N_4_, (**b**) UiO-66-NH_2_, (**c**) CdS, (**d**) gU-3, (**e**) gUS-1 and (**f**) EDS of gUS-1. TEM images of (**g**) gUS-1, (**h**) HRTEM image of gUS-1, and (**i**) EDX mapping of gUS-1.

The morphology of each as-synthesized photocatalyst was characterized by TEM and SEM (Figure 3). The g-C_3_N_4_, UiO-66-NH_2_, and CdS were found to be composed of sheets (Figure 3a), spherical particles (Figure 3b), and walnut-like nanoparticles (Figure 3c), respectively, all of which are consistent with literature reports [27,28,29]. When the UiO-66-NH_2_ was introduced, the sheet structure of the g-C_3_N_4_ transitioned to agglomerated particles (Figure 3d), possibly as a result of the formation of a heterostructure between the UiO-66-NH_2_ and g-C_3_N_4_. In addition, Figure 3e clearly shows CdS particles on the gUS-1 composite. EDS was used to confirm the presence of Cd and S in the gUS-1 specimen (Figure 3f) at concentrations of approximately 14 and 1.3 wt%. Figure 3g,h present TEM and high resolution TEM (HRTEM) micrographs of the gUS-1, which demonstrate that the UiO-66-NH_2_ nanoparticles were evenly dispersed over the g-C_3_N_4_ sheets (as indicated by the red rectangles in Figure 3g). The HRTEM image of the gUS-1 shows lattice spacings of 0.335 and 0.358 nm that correspond to the (002) and (100) planes of the CdS, respectively, confirming that CdS was successfully loaded onto the g-C_3_N_4_/UiO-66-NH_2_. The EDS map in Figure 3i demonstrates that Zr (yellow), Cd (violet), S (red), and N (green) were homogeneously dispersed on the g-C_3_N_4_ surface, suggesting that UiO-66-NH_2_ and CdS were grown on the g-C_3_N_4_ sheets.

**Figure 4:**
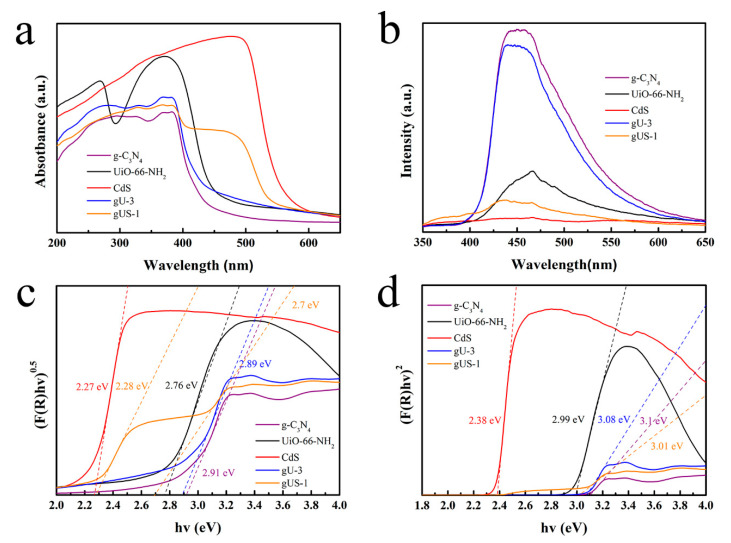
(**a**) UV-Vis DRS spectra obtained from the g-C_3_N_4_, UiO-66-NH_2_, CdS, gU-3, and gUS-1; (**b**) Photoluminescence (PL) spectra of the as-synthesized photocatalysts; (**c**) plot of (F(R)hv)^0.5^ as a function hν; (**d**) plot of (F(R)hv)^2^ as a function hν.

The optical properties of the g-C_3_N_4_, UiO-66-NH_2_, CdS, gU-3, and gUS-1 were assessed using UV–Vis and PL spectroscopy, with the results presented in Figure 4. The UiO-66-NH_2_ and g-C_3_N_4_ both absorbed strongly in the range of 200–450 nm (Figure 4a), and the estimated band gaps for these materials were 2.76 and 2.91 eV (Figure 4c), in agreement with literature values. The visible light absorption threshold of the gU-3 composites was also around 450 nm. Interestingly, the energy gap of each gU-3 composite (2.89 eV) was similar to that of the g-C_3_N_4_, implying that the g-C_3_N_4_ and UiO-66-NH_2_ were not simply mixed but rather formed a heterojunction [30]. The CdS was also found to absorb strongly around 580 nm (2.27 eV). After hybridization, the gUS-1 composites showed strong absorption in the range of 200–550 nm. The corresponding absorbance edges at 425 and 550 nm (2.7 and 2.28 eV) can possibly be attributed to the interactions between the CdS, UiO-66-NH_2_, and g-C_3_N_4_ [31]. Furthermore, the indirect band gap of g-C_3_N_4_, UiO-66-NH_2_, CdS, gU-3, and gUS-1 were 3.1, 2.99, 2.38, 3.08, and 3.01 eV, respectively (Figure 4d). These data also show that the presence of CdS increased the range of visible light that the g-C_3_N_4_/UiO-66-NH_2_/CdS was able to absorb, and so improved the catalyst’s utilization of visible light.

Figure 4b provides the PL spectra of the photocatalysts at an excitation wavelength of 325 nm. The powdered g-C_3_N_4_ exhibited strong photoluminescence, demonstrating the rapid recombination of photogenerated electron–hole pairs. The intensity of the gU-3 was lower, suggesting that the UiO-66-NH_2_ suppressed the recombination of photogenerated carriers. Moreover, the loading of CdS further reduced the photoluminescence intensity. The gUS-1 showed lower fluorescence intensity than the gU-3, indicating that the recombination rates of electron–hole pairs were inhibited in the former. The CdS had the lowest fluorescence intensity and so evidently had the highest separation rate of photogenerated electron–hole pairs. However, the CdS also showed poor photocatalytic activity, and so the photodegradation process appears to have been controlled by many factors. The g-C_3_N_4_/UiO-66-NH_2_ in the gUS-X appears to have had the primary effect during the photodegradation of TC. The above results indicate that, following the incorporation of the CdS, the light absorption of the material increased, which in turn promoted the photocatalytic reactions.

### 3.2. Photocatalytic Degradation of Tetracycline

#### 3.2.1. Effect of the CdS Loading Amount

**Figure 5:**
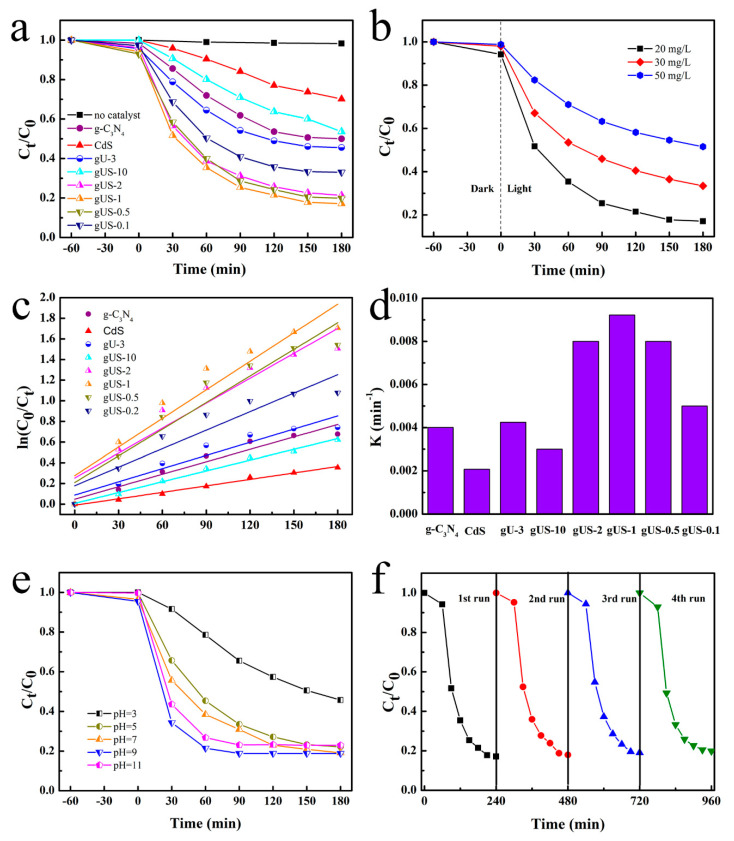
(**a**) Effect of the CdS loading amount, (**b**) effect of the initial tetracycline (TC) concentration on the performance of the gUS-1, (**c**) pseudo-first-order kinetics plots for the degradation of TC over these catalysts, (**d**) reaction rate constants for the various catalysts, (**e**) effect of initial pH on the degradation of TC over the gUS-1, and (**f**) recycling trials during the photocatalytic degradation of TC over the gUS-1.

The effect of CdS loading on the performance of the g-C_3_N_4_/UiO-66-NH_2_ was evaluated by assessing the photocatalytic activities during the degradation of TC over the g-C_3_N_4_, CdS, gU-3, and gUS-X. As a reference, a blank experiment was also carried out without a catalyst, using an initial TC concentration of 20 mg/L under visible light. This trial established that there was very little photolysis of the TC under a 300W xenon lamp after 3 h, indicating that TC was stable under these conditions and the rate of degradation was negligible. From Figure 5a, it is apparent that the TC degradation rates decreased in the order of gUS-X > gU-3 > g-C_3_N_4_ > CdS. The photocatalytic efficiencies of the composite photocatalysts were, therefore, higher than those of the single materials. Specifically, the as-prepared gUS-1 displayed the highest photocatalytic performance, with 83% TC decomposition. The TC removal provided by the gUS-1 was almost 30% higher than that of the gU-3, showing that the CdS played an important role in facilitating the overall photocatalytic process. The optimal CdS loading was evidently on the order of 50 wt%, and so the gUS-1 composite was used for further investigations. However, it should be noted that the removal efficiencies shown by the gUS-0.5 and gUS-2 were similar to that obtained from the gUS-1 (Figure 5a), meaning that CdS loadings in the range of 33 to 66 wt% provided a similar level of performance. Moreover, the photocatalytic performance declined with further increases in the CdS loading. This result indicates that the CdS is the not most important factor in the TC oxidation process, in agreement with the PL data.

#### 3.2.2. Effect of the Initial TC Concentration

The initial concentration of TC is a crucial factor affecting the removal efficiency [32,33,34], and so a series of photocatalytic degradation tests using the gUS-1 composite were carried out at different initial TC levels in the range of 20–50 mg/L. As shown in Figure 5b, these experiments were divided into two parts: an adsorption process (the dark part of experiments) and a photocatalytic process (the light part of experiments). The results show that the adsorption of TC by the gUS-1was unaffected by variations in the TC concentration, with removal percentages by adsorption of 5%, 2%, and 1%, respectively, at the different TC concentrations. This variation is considered to be negligible compared to the removal associated with the photocatalytic process. During the light stage, photodegradation percentages of 83%, 67%, and 50% were obtained at initial concentrations of 20, 30, and 50 mg/L, respectively. These correspond to degradation capacities of 33.2, 40.2, and 50 mg/g, respectively. It is evident that the removal capacity of the photocatalyst increased along with increases in the initial TC concentration, which reflects the increased difficulty in removing trace contaminants. The photocatalytic activity of the gUS-1 sample during TC degradation is compared with previously reported data in Table 1 [35,36,37,38,39]. This comparison demonstrates that the gUS-1 exhibited high removal efficiency relative to similar photocatalysts. Hence, the present g-C_3_N_4_/UiO-66-NH_2_/CdS photocatalysts could potentially be used to mitigate antibiotic contamination of water.

As can be seen from Figure 5c, the degradation data are well fit (with correlation coefficients of at least 0.87) using a pseudo-first-order kinetics model. Figure 5d shows that the gUS-1 exhibited the highest reaction rate constant for TC degradation (0.00922 min^−1^), and this value was 4.4 and 2.3 times those obtained for the CdS (0.00208 min^−1^) and g-C_3_N_4_ (0.00401 min^−1^). These results provide additional evidence that the g-C_3_N_4_/UiO-66-NH_2_/CdS was a more effective photocatalyst for TC degradation than the parent materials under visible light irradiation. The values of the kinetic parameters are set out in the Table 2.

#### 3.2.3. Effect of pH

In general, the pH of a solution is an important factor that affects the degradation efficiency during photocatalysis. In addition, TC is an amphoteric compound that takes different forms with changes in pH. According to the literature, TC is primarily in the form of a cation under acidic conditions (pH < 4), a zwitterion over the pH range of 4–7.5, and an anion in the pH range of 7.5–10 [40]. The present work performed trials over the pH span of 3–11 so as to assess each of these ranges, and the effect of pH on the degradation of TC using the gUS-1 sample is shown in Figure 5e. The pH of the solution had only a minimal effect on the adsorption capacity of the gUS-1, while the photodegradation efficiency was significantly modified by changes in the pH of the TC solution. Specifically, the photodegradation rate of TC roughly follows in the order of pH (9) > pH (11) > pH (7) > pH (5) > pH (3). As shown in Table 3, the maximum rate constant (k) increased with increases in the initial pH of the solution, suggesting that the photodegradation performance of the gUS-1 under neutral or alkaline conditions was enhanced. This finding that cationic TC inhibits the photo-oxidation process is in agreement with previous reports [41,42,43]. This phenomenon may be explained by the higher electron density in the ring system of anionic TC compared with the cation, which promotes the attack of radical species. In addition, the OH^−^ concentration in the reaction solution would be low at lower pH values, and this is not conducive to the additional generation of OH radicals.

#### 3.2.4. Reusability and Stability of the gUS-1 Composites

**Figure 6:**
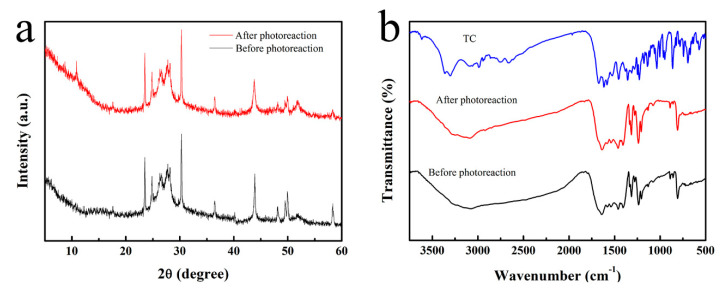
(**a**) XRD patterns and (**b**) FT-IR spectra obtained from the gUS-1 composite before and after four repeated of photocatalytic TC degradation trials.

The recycling characteristics of a photocatalyst are an important factor with regard to industrial applications, and so the stability of the gUS-1 during repeated uses was examined. After each use, the sample was collected by centrifugation, washed, and dried before the next degradation test. During four repeated trials, the photocatalytic performance of the material, as reflected in the TC degradation efficiency, remained constant at approximately 80.1% (Figure 5f), indicating that the gUS-1 had excellent recycling properties. Figure 6 displays the XRD patterns (a) and FT-IR spectra (b) acquired from the gUS-1 before and after the four cycles. There are no significant differences in the characteristic peak positions between before and after the replicate trials, confirming that the crystalline structure of the gUS-1 was not changed.

#### 3.2.5. Electrochemical Properties of gUS-1

**Figure 7:**
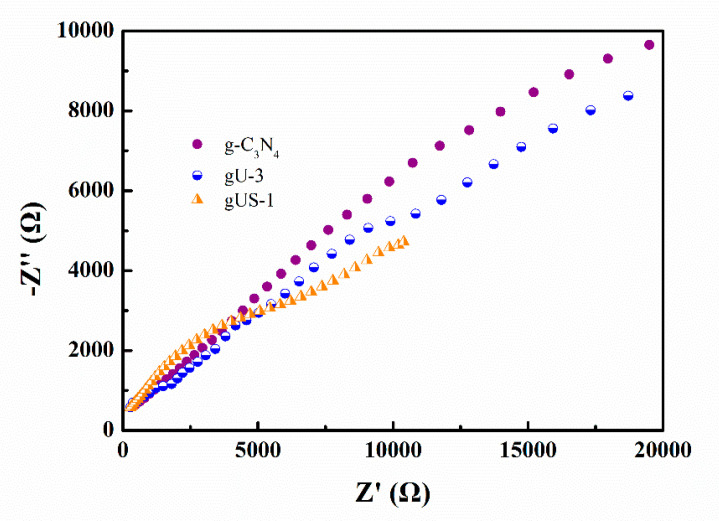
EIS data obtained from the g-C_3_N_4_, gU-3, and gUS-1 sample.

In general, a smaller arc radius in the electrochemical impedance spectroscopy (EIS) data for a semiconductor indicates a lower charge-transfer resistance. Figure 7 presents the EIS spectra obtained from the g-C_3_N_4_, gU-3, and gUS-1 samples. It is evident that the g-C_3_N_4_ produced the largest radius, suggesting that the transfer of photoinduced carriers was hindered by the higher charge-transfer resistance of g-C_3_N_4_. Conversely, the radius generated by the gUS-1was the smallest among these samples, meaning that the separation of photoinduced electron–hole pairs was more rapid, and the charge transfer efficiency in the g-C_3_N_4_/UiO-66-NH_2_/CdS photocatalysts was improved. These results are in good agreement with those obtained from the PL analyses.

### 3.3. Possible Mechanism of Photocatalytic Degradation

**Figure 8:**
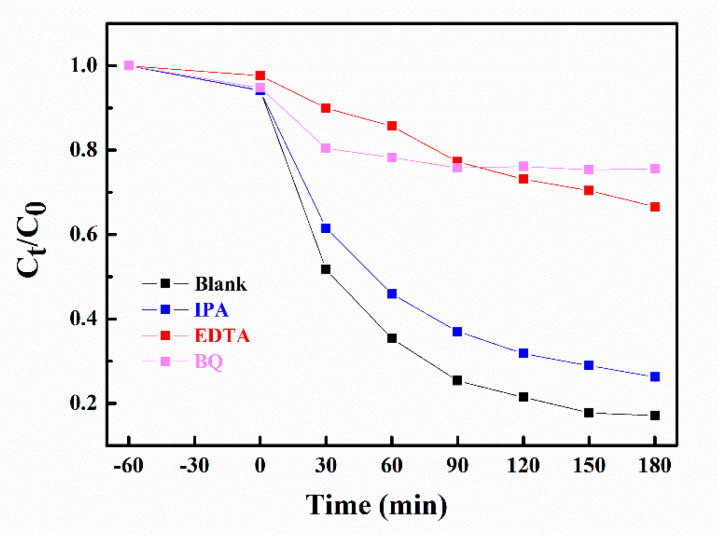
Active species trapping data acquired from TC degradation under visible light irradiation over the gUS-1 composite.

Hydroxyl radicals (⋅OH), superoxide radicals (⋅O^2−^), and holes (h^+^) are critical factors in photocatalytic oxidation [44]. Thus, to better understand the role of different free radicals in the degradation of TC, radical capture experiments were carried out. In these trials, benzoquinone (BQ), isopropanol (IPA), and ethylenediaminetetraacetic acid disodium salt (EDTA-2Na) were used to scavenge ⋅O^2−^, ⋅OH, and h^+^, respectively [45]. As can be seen from Figure 8, the photocatalytic activity significantly decreased with the addition of BQ and EDTA-Na, suggesting that h^+^ and ⋅O^2−^ were the major active species in the photocatalytic process using gUS-1. The addition of IPA had little effect on the degradation efficiency, and so ⋅OH was evidently a secondary active species. Thus, both ⋅O^2−^ and h^+^ are believed to have been the primary active species during the photocatalytic degradation of TC over the g-C_3_N_4_/UiO-66-NH_2_/CdS photocatalysts.

**Figure 9:**
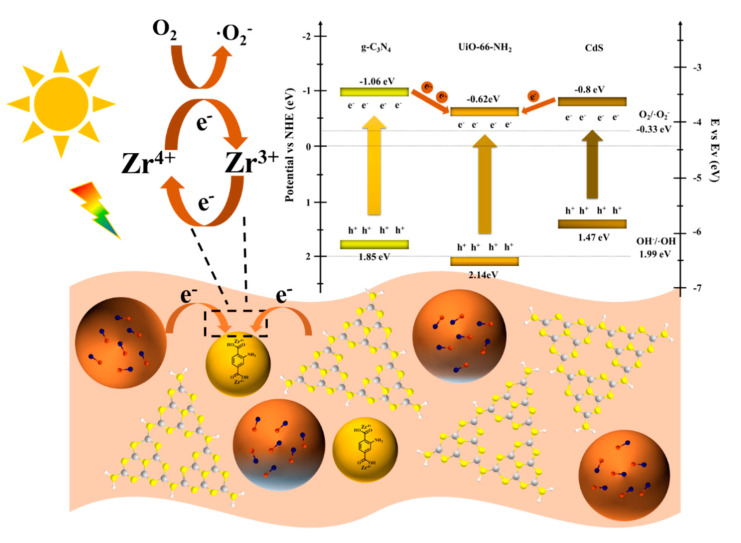
Proposed photocatalytic degradation mechanism.

Given the above experiment results, a plausible photocatalytic mechanism for TC degradation over the gUS-1 composite was devised and is summarized in Figure 9. Note that UPS data and the band structure of photocatalyst are provided in Figure S1 and Table S1. Because the conduction band (CB) potentials of the g-C_3_N_4_ and CdS were more negative than that of the UiO-66-NH_2_, electrons from the CB of the former materials could be injected into the lowest unoccupied molecular orbital (LUMO) of the UiO-66-NH_2_, resulting in the effective inhibition of photogenerated electron–hole pair recombination. Accumulated electrons in the UiO-66-NH_2_ with a potential lower than −0.62 eV were then capable of reducing dissolved oxygen adsorbed onto the gUS-1 surface to generate ⋅O^2−^ (E(O_2_/⋅O_2_^−^) = −0.33 eV vs. NHE) [46]. In this process, photoelectrons were transferred to Zr-O clusters to produce Zr^3+^ ions and could react with O_2_ to generate ⋅O^2−^, while the Zr^3+^ ions were oxidized back to Zr^4+^ [47,48,49]. Simultaneously, the h^+^ in the valence band (VB) of UiO-66-NH_2_ migrated to the VB of the g-C_3_N_4_ or CdS, such that only a small proportion of the h^+^ located in the UiO-66-NH_2_ VB generated ·OH radicals. The photogenerated h^+^ in the VB of the CdS or g-C_3_N_4_ were incapable of oxidizing hydroxyl groups into ⋅OH radicals (E(OH^−^/⋅OH) = +1.99 eV vs. NHE) [50], and so these accumulated h^+^ instead oxidized the TC. Therefore, both h^+^ and ⋅O^2−^ played primary roles in the photodegradation of TC, in agreement with the experimental result. Furthermore, as noted, introducing the CdS extended the range of light absorption and improved the separation of electron–hole pairs. The overall photocatalytic reaction sequence over the gUS-1 during TC degradation can, therefore, be described by the series of equations:

g-C_3_N_4_/CdS + hv → e^−^ +h^+^,

UiO-66-NH_2_(Zr^4+^) + e^−^ → UiO-66-NH_2_(Zr^3+^),

UiO-66-NH_2_(Zr^3+^) + O_2_ → UiO-66-NH_2_(Zr^4+^) +⋅O^2−^,

h^+^/⋅O^2−^ + TC → degraded products.

## 4. Conclusions

A g-C_3_N_4_/UiO-66-NH_2_/CdS composite catalyst was prepared by a combination of calcination and hydrothermal methods, and the gUS-1 specimen demonstrated the best photocatalytic performance with regard to the degradation of TC under visible light. Compared with the UiO-66-NH_2_ and g-C_3_N_4_, the photoabsorption region of the composite material was widened, while its fluorescence intensity was diminished. The experimental data also indicate that the photocatalytic activity of the gUS-1 during the oxidation of TC was higher than that of the CdS or g-C_3_N_4_ under visible light, and that the former material provided a reaction rate constant 4.4 and 2.3 times those associated with TC degradation over the CdS and g-C_3_N_4_, respectively. This enhanced photocatalytic activity is ascribed to the loading of CdS, which improved the utilization of visible light and restrained the recombination of the electrons and the holes. This work provides a new theoretical background for the design of novel photocatalytic materials for the treatment of antibiotic-contaminated wastewaters.

## Figures and Tables

**Table 1:** Comparison of the photocatalytic TC degradation of various photocatalysts.

Samples	C_Catalyst_ g/L	C_TC_ mg/L	DR%	References
Sn_3_O_4_/g-C_3_N_4_	0.5	10	72.2%	35
g-C_3_N_4_/Nb_2_O_5_	0.5	10	76.2%	36
β-Bi_2_O_3_@g-C_3_N_4_	0.5	10	80.2%	37
g-C_3_N_4_/Ag_2_CO_3_/graphene	0.6	20	81.6%	38
g-C_3_N_4_/Ag/P3HT	1	20	75%	39
gUS-1	0.5	20	83%	this work

**Table 2:** The pseudo-first-order reaction kinetics parameters for the various catalysts.

Sample	k	b	R^2^
g-C_3_N_4_	0.00401	0.04827	0.94
CdS	0.00208	−0.01071	0.99
gU-3	0.00425	0.08814	0.90
gUS-10	0.003	0.007	0.99
gUS-2	0.008	0.252	0.89
gUS-1	0.00922	0.27568	0.89
gUS-0.5	0.008	0.209	0.90
gUS-0.1	0.005	0.177	0.87

**Table 3:** Pseudo-first-order reaction kinetics parameters for TC degradation at various pH values.

pH	k	b	R^2^
3	0.00458	−0.00162	0.99
5	0.00846	−0.01071	0.93
7	0.00872	0.27789	0.91
9	0.01781	0.27994	0.82
11	0.01625	0.17306	0.87

## Supplementary Materials

The following are available online at https://www.mdpi.com/2079-4991/10/9/1824/s1. Figure S1: UPS spectra of g-C_3_N_4_, UiO-66-NH_2_, and CdS; Table S1: band structures of the g-C_3_N_4_, UiO-66-NH_2_, and CdS.
