# Supplementary material for: Preparation of a g-C3N4/UiO-66-NH2/CdS Photocatalyst with Enhanced Visible Light Photocatalytic Activity for Tetracycline Degradation
Source: Nanomaterials (Basel). 2020 Sep 12;10(9):1824. doi: 10.3390/nano10091824 (PMC7558207; doi:10.3390/nano10091824)
Supplement: Supplementary file 1 [file nanomaterials-10-01824-s001.pdf]

Supplementary Materials

Preparation of a g-C_3N_4/UiO-66-NH_2/CdS Photocatalyst with Enhanced Visible Light Photocatalytic Activity for Tetracycline Degradation

Hao Zhang [1,2], Jialiang Li [1,*], Xianglei He [1] and Bo Liu [2,*]

[1] School of Chemistry and Chemical Engineering, Shandong University of Technology, Zibo 255000, China; haozhangy@163.com (H.Z.); xianglei_he@163.com (X.H.)

[2] Laboratory of Functional Molecules and Materials, School of Physics and Optoelectronic Engineering, Shandong University of Technology, Zibo 255000, China

* Correspondence: lijialiang@sdut.edu.cn (J.L.); liub@sdut.edu.cn (B.L.)

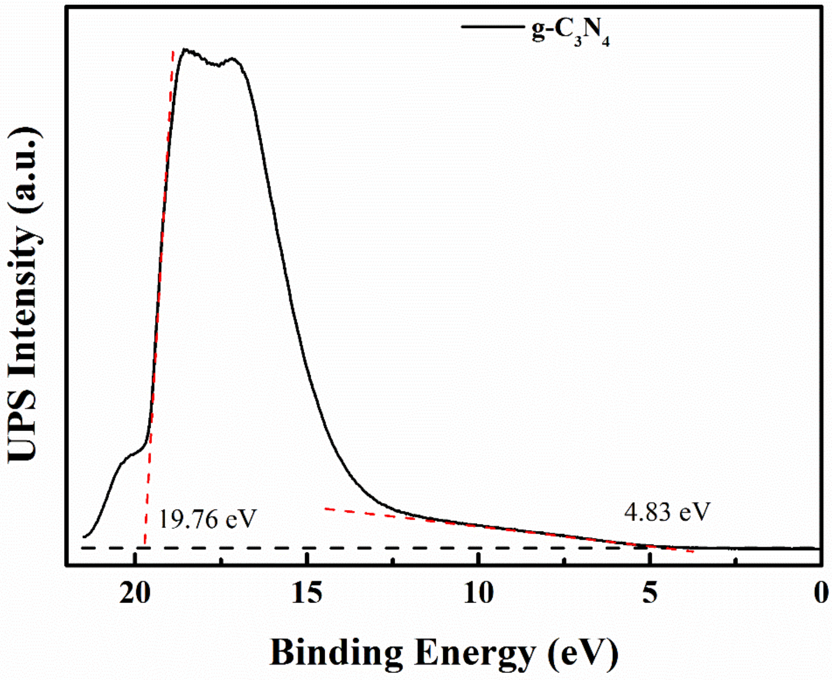

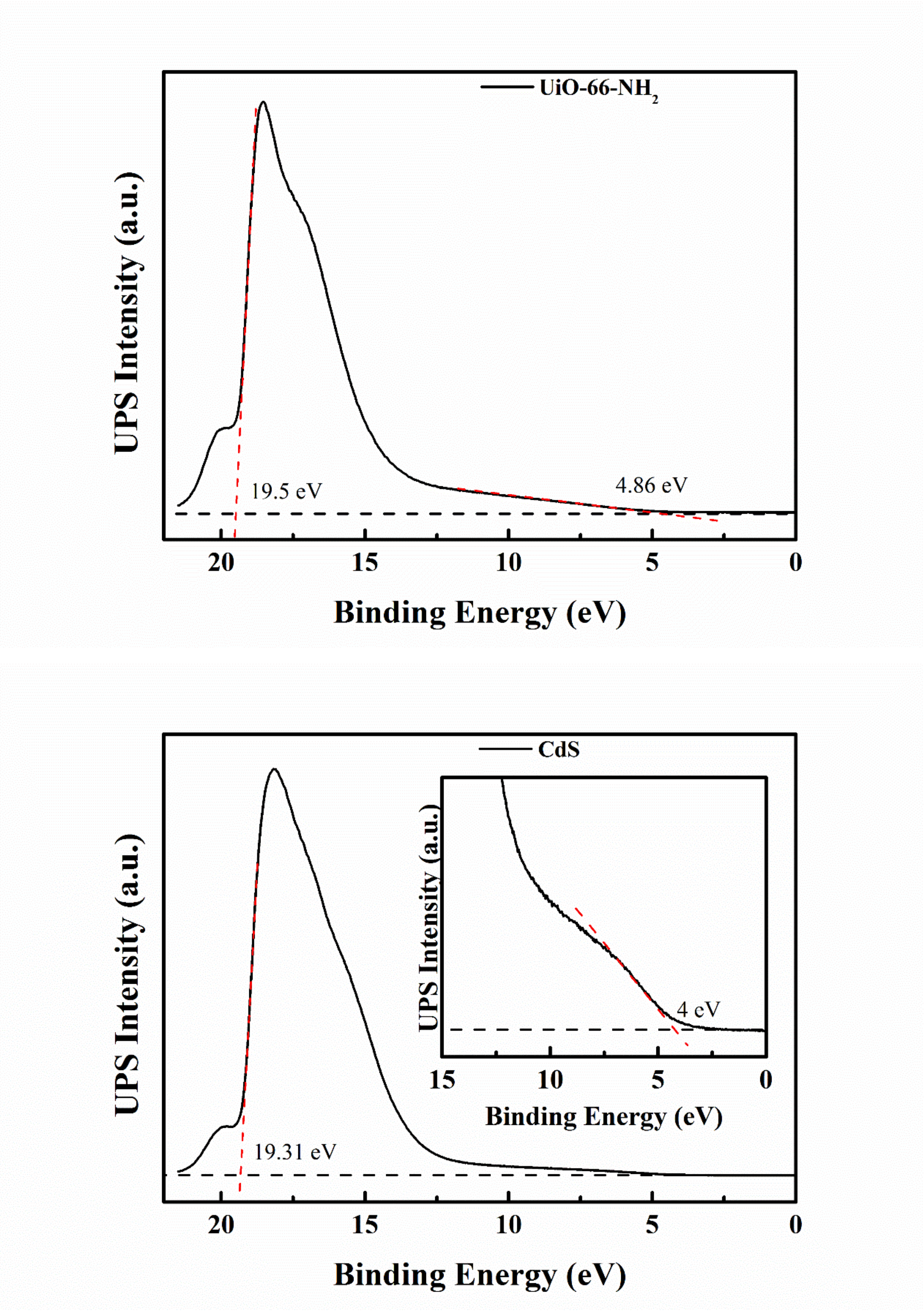

Figure S1. UPS spectra of g-C_3N_4, UiO-66-NH_2 and CdS

Table 1. Band structure of g-C_3N_4, UiO-66-NH_2 and CdS.

Sample	Eg (eV)	HBE	LBE	hv	Ee	E_{VB}	E_{CB}
**g-C_3N_4**	2.91	19.76	4.83	21.22	4.44	1.85	−1.06
**UiO-66-NH_2**	2.76	19.5	4.86	21.22	4.44	2.14	−0.62
**CdS**	2.27	19.31	4	21.22	4.44	1.47	−0.8
